# Unveiling new protein biomarkers and therapeutic targets for acne through integrated analysis of human plasma proteomics and genomics

**DOI:** 10.3389/fimmu.2024.1452801

**Published:** 2024-10-18

**Authors:** Sui Deng, Rui Mao, Yifeng He

**Affiliations:** ^1^ Changde Hospital, Xiangya School of Medicine, Central South University (The First People’s Hospital of Changde City), Changde, China; ^2^ Department of Dermatology, Xiangya Hospital, Central South University, Changsha, China

**Keywords:** drug target, genetics, Mendelian randomization, plasma proteomics, prospective studies, acne

## Abstract

**Background:**

The current landscape of acne therapeutics is notably lacking in targeted treatments, highlighting a critical need for the discovery of new drug targets to improve treatment outcomes.

**Objectives:**

This study aims to investigate the connections between proteomics and genetics in relation to acne across extensive population cohorts, aspiring to identify innovative preventive and therapeutic approaches.

**Methods:**

Employing a longitudinal cohort of 54,306 participants from the UK Biobank Pharmacological Proteomics Project (UKB-PPP), we performed an exhaustive evaluation of the associations between 2,923 serum proteins and acne risk. Initial multivariate Cox regression analyses assessed the relationship between protein expression levels and acne onset, followed by two-sample Mendelian Randomization (TSMR), Summary-data-based Mendelian Randomization (SMR), and colocalization to identify genetic correlations with potential protein targets.

**Results:**

Within the UKB cohort, we identified 19 proteins significantly associated with the risk of acne. Subsequent analysis using Two-Sample Mendelian Randomization (TSMR) refined this to two specific proteins: FSTL1 and ANXA5. Each one-standard deviation increase in the expression levels of FSTL1 and ANXA5 was associated with a 24% and 32% increase in acne incidence, respectively. These results were further validated by additional Summary-data-based Mendelian Randomization (SMR) and differential expression analyses.

**Conclusions:**

Our comprehensive analysis of proteomic and genetic data from a European adult cohort provides compelling causal evidence that several proteins are promising targets for novel acne treatments.

## Introduction

The prevalence of acne remains significant (1, 2). The underlying mechanisms of acne are complex and not yet fully understood. Current standard treatments, largely reliant on immunosuppressants and nonspecific anti-inflammatory medications, provide only symptomatic relief. Such treatments can mitigate symptoms but often do not prevent recurrence, imposing significant physical and psychological burdens on patients, in addition to substantial economic and healthcare costs. This scenario underscores the critical need for the identification and development of novel therapeutic targets (3, 4). Advancing our understanding and treatment modalities for acne is imperative to enhance patient outcomes, reduce healthcare burdens, and address the limitations and risks associated with current treatment options.

The circulating proteome represents a dynamic network encompassing all blood proteins, mirroring an individual’s genetic predispositions and external influences, including environmental exposures and lifestyle changes. Focused analyses of select biomarkers have demonstrated that the confluence of proteomic and genetic data can elucidate causal links between proteins and diseases, thereby uncovering viable drug targets within the bloodstream (5, 6). The potential of broad, unbiased population-level analysis of the circulating proteome to yield novel insights into the biology of diseases, particularly in enhancing the understanding, treatment, and prognosis of prevalent acne, is yet to be fully determined.

In our research, we leveraged a longitudinal cohort comprising 54,306 participants from the UKB-PPP to perform an extensive analysis of both observed and genetic correlations between 2,923 proteins and the susceptibility to prevalent acne. Utilizing single-cell transcriptome data, we delved into the expression patterns of identified drug targets and investigated potential mechanisms underlying the pathogenesis of acne.

## Methods

### Study design and participants

**Figure 1:**
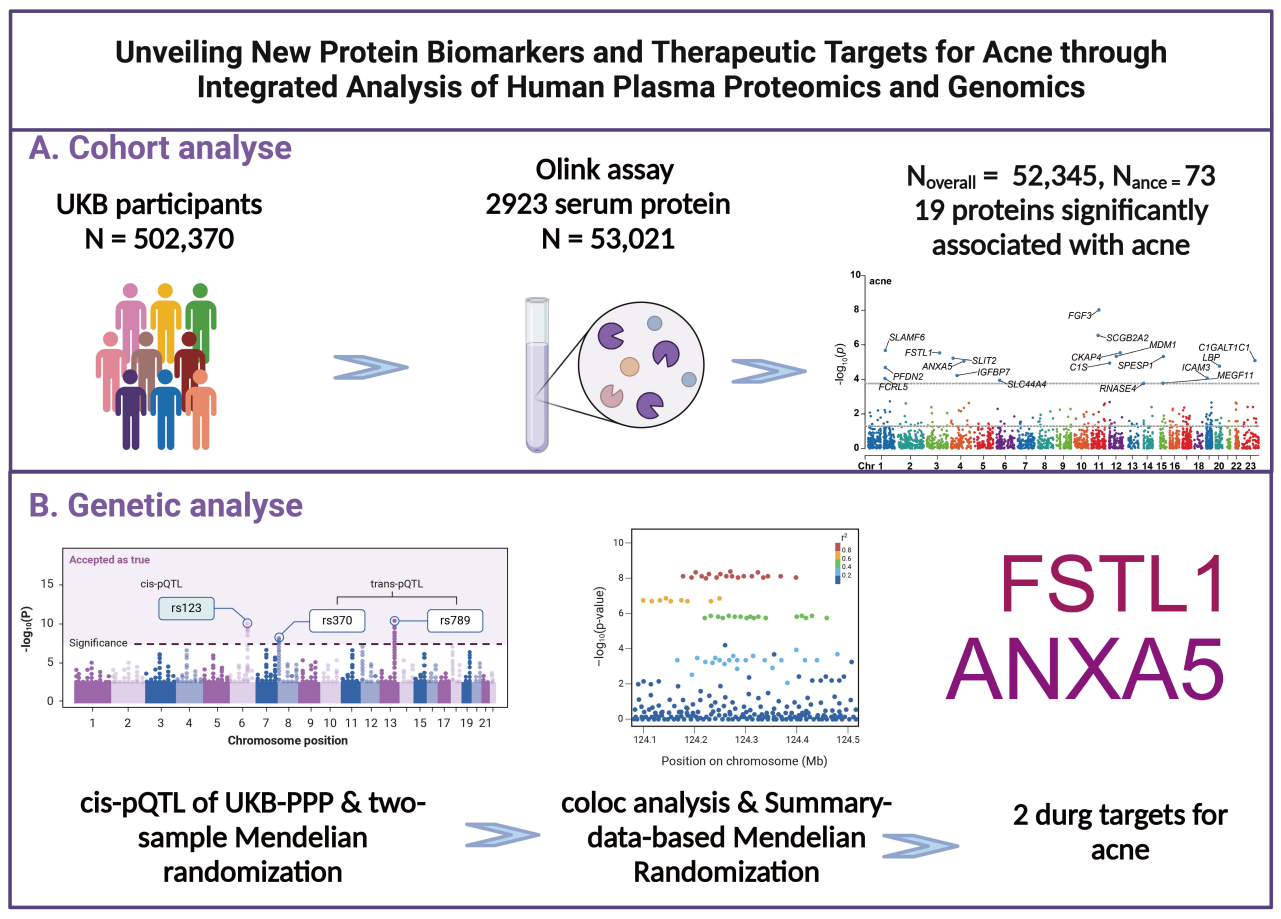
Research design and flowchart. **(A)** Process for Prospective Cohort Analysis **(B)** Processes for Mendelian Randomization, Colocalization, and Additional Genetic Analyses.

The study’s methodology is depicted in **Figure 1**. The UK Biobank constitutes a population-based cohort, comprising approximately 502,370 volunteers aged between 40 and 69 years at the inception of the study (7). These participants were recruited from 22 assessment centers throughout the UK during 2006-2010. Upon enrollment, individuals provided informed consent, underwent physical examinations, and supplied comprehensive data on socio-demographic attributes, lifestyle habits, medical histories, medication usage, in addition to blood samples. Participant events were meticulously tracked via electronic health records up to July 2023.

The UKB-PPP, a collaborative effort funded by 13 biopharmaceutical companies, was established to generate blood-based proteomic data for a subset of the UK Biobank cohort (7, 8). This endeavor encompassed 54,306 participants, with 46,673 (85.9%) being randomly chosen from the initial cohort, 6,385 (11.8%) pre-selected by consortium members based on specific criteria (such as disease status or genetic lineage), and 1,268 (2.3%) selected due to their involvement in multiple COVID-19 case-control imaging studies. Following rigorous quality control measures, data from 53,021 participants were deemed suitable for inclusion in our analysis (as shown in **Figure 1**). Ethical approval for the UK Biobank was granted by the North West Multicenter Research Ethics Committee. All procedures and analyses conducted were in strict accordance with the stipulations of UK Biobank Application Number 93810.

### Protein quantification and the processing of proteomic data within the UK Biobank

UKB-PPP have been elaborated in prior publications (9). In summary, blood specimens collected from participants at the commencement of the UKB-PPP were subjected to analysis via the Olink Explore 1536 platform (Olink Proteomics, Inc.; Waltham, MA). This platform employs proximity extension assay (PEA) technology for the quantification of 2,923 protein analytes across four distinct protein panels (**Supplementary Table 1**). Due to significant data omission rates, three proteins—GLIPR1 (99.7%), NPM1 (74.0%), and PCOLCE (63.6%)—were omitted from the final cohort analysis. For the assessment of the remaining proteins, the k-nearest neighbors (k-NN) algorithm (with k = 10) was utilized to impute missing data within the proteome dataset (10). Subsequently, Z-score normalization was applied to all 2,920 protein markers prior to their inclusion in the analysis.

### Assessment of outcome

The ascertainment of outcomes was primarily based on hospital records sourced from the Hospital Episode Statistics in England, the Patient Episode Database for Wales, and the Scottish Morbidity Records. This was supplemented by self-reported diagnoses, primary care records, and death registry data. Diagnoses of acne (ICD-10: L70) was documented utilizing the International Classification of Diseases (ICD) Coding System. Participants who had self-reported these conditions or had been diagnosed through hospital admissions at the start of the study were excluded from the analysis. Follow-up for each participant extended from their initial assessment date to July 1, 2023, ensuring a comprehensive longitudinal analysis of health outcomes.

### Proteomic association analyses

Primary analyses were conducted to investigate the relationships between circulating protein levels and the incidence of acne utilizing Cox proportional hazards models. These models incorporated adjustments for several covariates to account for potential confounding factors. Adjustments included age (treated as a sequential variable), gender (male/female), body mass index (BMI, calculated as kilograms per square meter and treated as a continuous variable), and average annual household gross income (categorized into <£18,000, £18,000-£30,999, £31,000-£51,999, £52,000-£100,000, >£100,000, and “not known” or missing). Additionally, race (categorized as mixed-European, white, South Asian, black, and others), education level (ranging from CSEs or equivalent to professional qualifications, with an option for ‘None of the above’), smoking status (current, former, or never), and alcohol consumption frequency (never, occasionally, sometimes, or daily) were included as covariates. The Townsend Deprivation Index, a measure of socio-economic status, was included as a continuous variable. Physical activity levels were quantified in metabolic equivalent (met-minutes) per week and treated as a continuous variable, and the season of blood collection was categorized into winter (December–February), spring (March–May), summer (June–August), and autumn (September–November).

### Mendelian randomization analysis

This research strictly follows the “Strengthening the Reporting of Observational Studies in Epidemiology-Mendelian Randomisation (STROBE-MR) (11)” guidelines, outlined in **Supplementary Table 2**. The data utilized in this study, derived from genome-wide association studies (GWAS) and protein Quantitative Trait Loci (pQTL), were obtained solely from open-access databases with prior ethical approval. Detailed information about all the datasets employed in this analysis is available in **Supplementary Table 3**. In our investigation, plasma protein levels in UKB-PPP were established as the exposure variable, with acne defined as the outcome, utilizing the “TwoSampleMR” package for Mendelian Randomization (MR) analysis. For proteins represented by a single pQTL, the Wald ratio method was employed. In contrast, for proteins with multiple genetic instruments, the inverse variance-weighted MR (MR-IVW) method was applied. The odds ratio (OR) representing the increased risk of outcome correlates with a rise in one standard deviation (SD) of plasma protein levels.

We recognize the importance of validation across diverse cohorts and have accordingly utilized multiple significant pQTL datasets to affirm our results. To this end, we sourced summary-level genetic association data from the DECODE study involving 35,559 Icelandic participants, which analyzed 4,907 circulating proteins (12). Additionally, we included data from Pietzner et al. (13), which involved a cohort of 10,708 Finnish participants analyzing 4,775 plasma proteins. Further validation was carried out using the Meta cohort compiled by Zheng et al. (14), which amalgamated data from five previous GWAS (15–19), identifying 738 cis-acting SNPs linked to 734 proteins.

For an instrumental variable to be deemed valid, it must satisfy three essential criteria, as illustrated in **Supplementary Figure 1**: First, the genetic variant must have a strong association with the exposure. This was verified by calculating the instrument’s F-statistic, using the formula of the squared beta coefficient divided by the squared standard error, aiming for an F-statistic greater than 10 to avoid weak instrument bias. Second, the genetic variants must be independent of any confounders that could affect the exposure-outcome relationship. Through the PhenoScanner database (20), we identified any traits, other than the exposure, significantly associated with inflammatory skin disease (at P < 5×10^-8^). Considering literature, factors such as inflammatory bowel disease (21), and depression (22) have been identified as acne risk factors. Variants linked with these confounders were excluded to minimize horizontal pleiotropy. Third, the genetic variants are expected to affect the outcome solely through their impact on the exposure. To address confounding by population stratification, analyses were concentrated on individuals of a consistent ancestry. Furthermore, to tackle potential confounding by linkage disequilibrium, SMR and HEIDI analysis was conducted to assess the likelihood of genetic confounding.

We further implemented Steiger filtering to validate the directionality of associations between proteins and outcomes (23). A P-value threshold of less than 0.05 was deemed indicative of statistical significance. Detailed methods for analysis such as SMR, Bayesian co-localization, Phenome-Wide MR, and Single-cell RNA Sequencing Data Analysis are presented in the eMethods section of Supplementary 2 (24–36).

### Statistical analysis

In our preliminary analysis examining the association between the plasma proteome and acne, we applied the Benjamini-Hochberg (BH) procedure to adjust P values for multiple testing. This approach controls the false discovery rate (FDR), with an FDR threshold of less than 0.05 being interpreted as statistically significant. In the follow-up confirmatory MR analysis, we adhered to a criterion where P values less than 0.05 were deemed to indicate statistical significance. This standard threshold was used to establish a robust level of confidence in the observed associations, ensuring that the findings from the MR analysis are both reliable and indicative of genuine biological effects. Details of drug target development are categorized for clinical outcomes (Tclin) and chemical outcomes (Tchem). Additionally, information on drug targets, including their evaluation in phase II-IV clinical trials, is extensively sourced from the DrugBank and OpenTargets databases. All computational and statistical analyses in this study were performed using the R software (version 4.3.1) on a LINUX (CentOS) operating system. The employed packages encompassed ‘TwoSampleMR (version 0.5.6)’, ‘coloc (version 5.1.0.1)’, ‘SMR (version 1.3.1)’, ‘phenoscanner (version 1.2.2)’, and ‘CMplot (version 4.3.1)’.

## Results

The study encompassed 53,016 participants from the UKB-PPP, with their demographics and characteristics detailed in **Table 1**. The cohort was predominantly female (28,581 participants [53.9%]) and overwhelmingly white (49,010 participants [92%]), with an average age of 56.8 years (standard deviation [SD] = 8.2 years). Throughout the follow-up period, which lasted a median of 14.2 years (interquartile range [IQR] = 13.5-15 years), 5,3016 participants (1.4%) reported event of acne.

**Table 1:** Baseline characterization of participants in UKB.

Variable	Overall, N = 53,016	female, N = 28,581	male, N = 24,435	p-value^1^
**Age_when_attended, Mean (SD)**	56.81 (8.21)	56.61 (8.10)	57.04 (8.33)	<0.001
**BMI, Mean (SD)**	27.47 (4.80)	27.16 (5.22)	27.85 (4.24)	<0.001
**smoking_status, n (%)**				<0.001
current	5,637 (11%)	2,559 (9.0%)	3,078 (13%)	
previous	18,568 (35%)	9,027 (32%)	9,541 (39%)	
never	28,811 (54%)	16,995 (59%)	11,816 (48%)	
**Alcohol_intake_frequency, n (%)**				<0.001
never	4,604 (8.7%)	2,888 (10%)	1,716 (7.0%)	
occasionally	12,058 (23%)	8,097 (28%)	3,961 (16%)	
sometimes	25,656 (48%)	13,041 (46%)	12,615 (52%)	
Daily	10,698 (20%)	4,555 (16%)	6,143 (25%)	
**Summed_MET, Mean (SD)**	2,651.18 (2,653.68)	2,567.43 (2,510.17)	2,749.14 (2,809.15)	0.001
**Townsend, Mean (SD)**	-1.18 (3.19)	-1.22 (3.12)	-1.13 (3.26)	0.2
**education, n (%)**				<0.001
College or University degree	17,025 (32%)	8,804 (31%)	8,221 (34%)	
A levels/AS levels or equivalent	5,839 (11%)	3,379 (12%)	2,460 (10%)	
O levels/GCSEs or equivalent	10,994 (21%)	6,535 (23%)	4,459 (18%)	
CSEs or equivalent	2,837 (5.4%)	1,546 (5.4%)	1,291 (5.3%)	
NVQ or HND or HNC or equivalent	3,518 (6.6%)	1,307 (4.6%)	2,211 (9.0%)	
Other professional qualifications	2,813 (5.3%)	1,715 (6.0%)	1,098 (4.5%)	
None of the above	9,383 (18%)	4,997 (17%)	4,386 (18%)	
Prefer not to answer	607 (1.1%)	298 (1.0%)	309 (1.3%)	
**income, n (%)**				<0.001
Less_than_18,000	13,848 (26%)	7,942 (28%)	5,906 (24%)	
18,000_to_30,999	13,975 (26%)	7,781 (27%)	6,194 (25%)	
31,000_to_51,999	12,956 (24%)	6,852 (24%)	6,104 (25%)	
52,000_to_100,000	9,659 (18%)	4,776 (17%)	4,883 (20%)	
Greater_than_100,000	2,578 (4.9%)	1,230 (4.3%)	1,348 (5.5%)	
**race, n (%)**				<0.001
White	49,010 (92%)	26,371 (92%)	22,639 (93%)	
Asian_or_Asian_British	1,921 (3.6%)	1,162 (4.1%)	759 (3.1%)	
Black_or_Black_British	319 (0.6%)	163 (0.6%)	156 (0.6%)	
mixed	966 (1.8%)	437 (1.5%)	529 (2.2%)	
Chinese	152 (0.3%)	91 (0.3%)	61 (0.2%)	
Other ethnic group	648 (1.2%)	357 (1.2%)	291 (1.2%)	
**acne, n (%)**	744 (1.4%)	495 (1.7%)	249 (1.0%)	<0.001

^1^Wilcoxon rank sum test; Pearson’s Chi-squared test.

### Observational associations of proteins with acne

**Figure 2:**
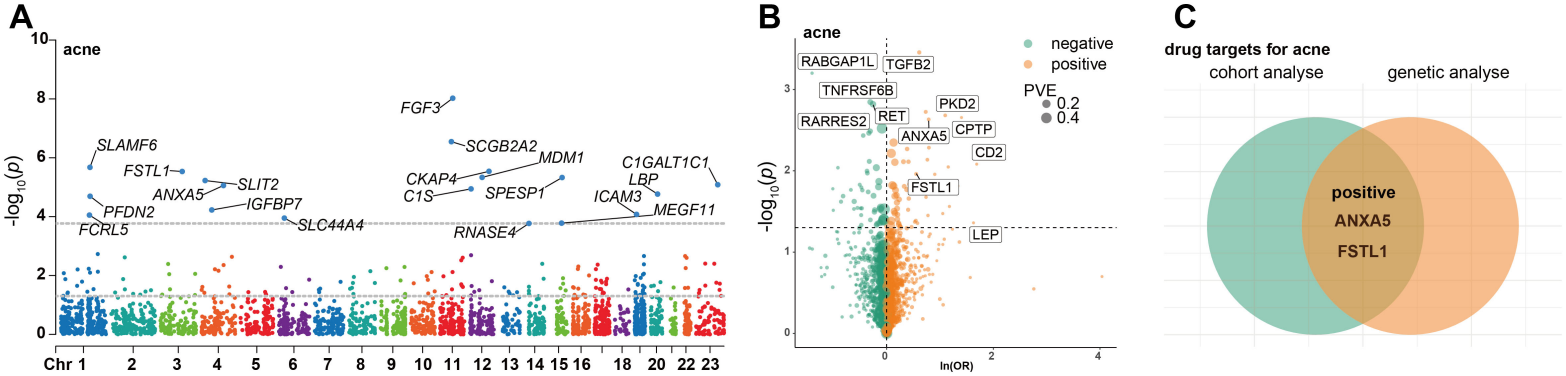
Discovery of Potential Drug Targets for Acne Through Integrative Cohort and Mendelian Randomization Analyses. This figure presents a comprehensive visualization of our analytical approach, starting with a Manhattan plot **(A)** detailing the cohort analysis outcomes. Each point on the plot signifies a distinct protein, with the x-axis denoting the gene's chromosomal location and the y-axis displaying the p-value obtained from multivariate Cox regression analysis. The region between the two dotted lines adjacent to the x-axis delineates the significance threshold (p < 0.05). Points beyond this threshold indicate proteins whose adjusted p-values remain below 0.05 after false discovery rate (FDR) correction, highlighted in blue and annotated with their respective protein names. Adjacent to this, a volcano plot **(B)** illustrates the results from cis-Mendelian Randomization (cis-MR) analysis, plotting each protein by the effect size from MR analysis (x-axis) against its p-value (y-axis). Lastly, a Venn diagram **(C)** captures the overlap between significant findings in both cohort and cis-MR analyses, with the names of the intersecting proteins specified within the diagram.

Following adjustments for age, gender, body mass index (BMI), average annual household gross income, race, education level, smoking status, alcohol consumption frequency, Townsend Deprivation Index, physical activity levels, and the season of blood collection, the results from the multivariable Cox regression model revealed that 19 proteins were significantly associated with the onset of acne, taking into account multiple corrections for the false discovery rate (FDR) (as illustrated in **Figure 2** and **Supplementary Table 5**, FDR < 0.05).

### Genetic associations of proteins with acne

**Figure 3:**
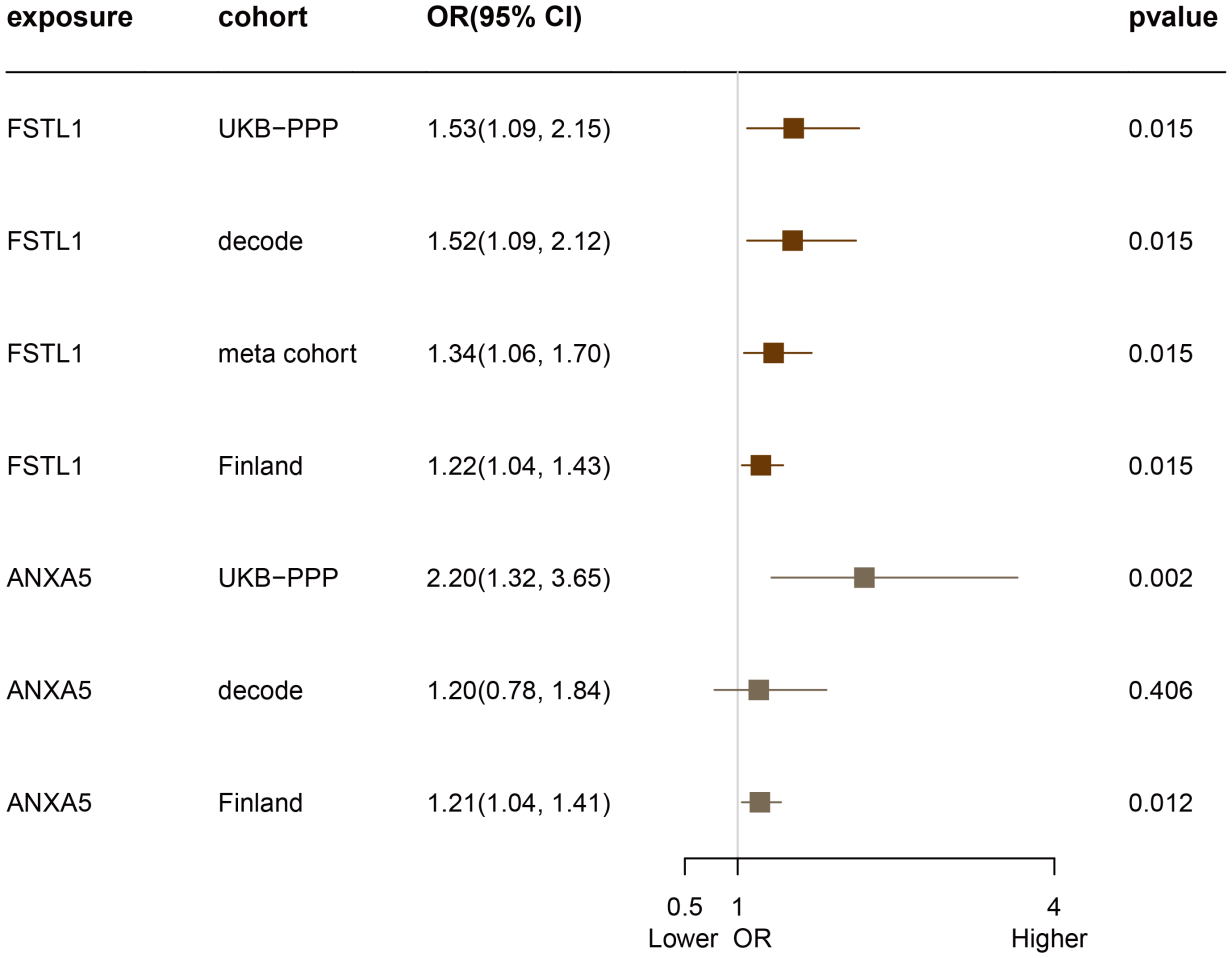
Evaluation of the Causal Relationship Between FSTL1 and ANXA5 Proteins and Acne Across Four Proteome Cohorts.

**Figure 4:**
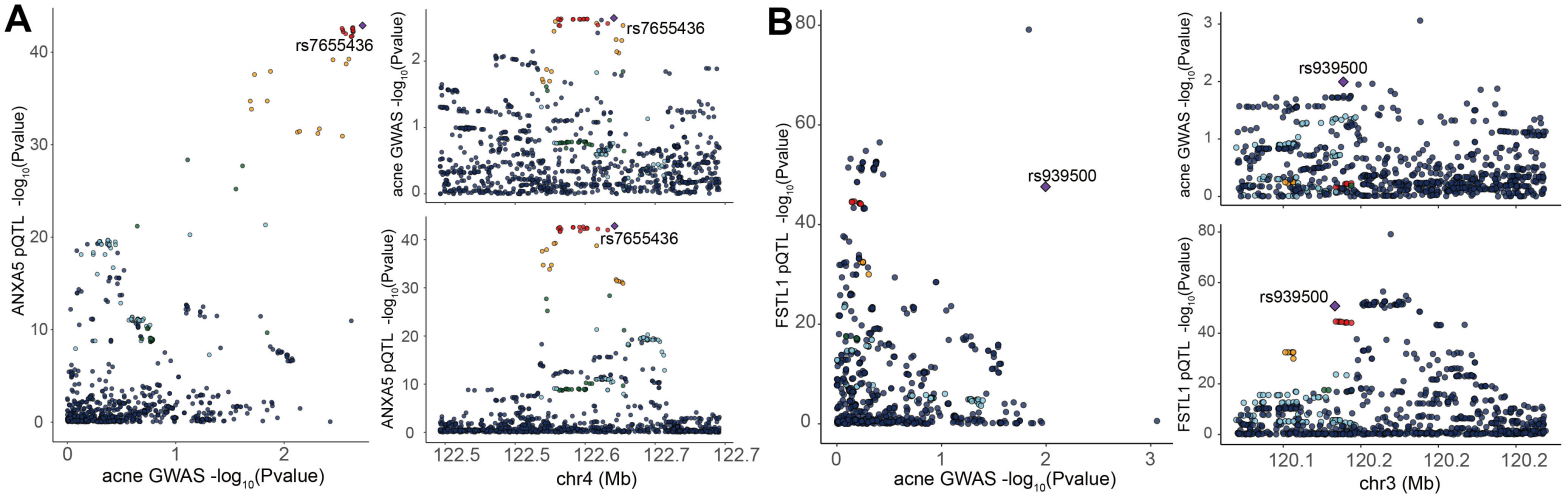
Bayesian Colocalization Analysis to Unveil Potential Causal Links Between Specific Plasma Proteins and Acne. Analysis of the ANXA5 **(A)**, FSTL1 **(B)**, and Acne: Illustrates colocalization analysis results for the plasma protein in the context of acne. Purple diamond-shaped markers denote single nucleotide polymorphisms (SNPs) that exhibit the lowest combined p-value from both the genome-wide association studies (GWAS) for the protein and the GWAS for traits related to acne.

Subsequent cis-MR analysis was conducted to assess the causal impact of identified proteins on acne. From the 2,920 proteins analyzed in the UKB-PPP, 2,030 cis-acting protein quantitative trait loci (cis-pQTLs) were identified (detailed in **Supplementary Table 6**). Among 19 proteins highlighted by the Cox analysis, 15 possessed cis-pQTLs with F statistics exceeding 10, indicating robust instrument strength. Through MR analysis and examination of causality directions, 2 proteins (FSTL1 and ANXA5) were causally implicated in acne out of the 14 analyzed (illustrated in **Figure 2**, **Table 2**, **Supplementary Table 7**). Furthermore, these two proteins’ significant associations were corroborated in the validation dataset (**Figure 3**). Lastly, phenotypic analysis revealed that the significant cis-pQTLs of proteins causally linked to acne showed no association with inflammatory bowel disease, depression, or other known risk factors for acne (**Supplementary Table 8**). SMR analysis revealed significant causal associations between the expression levels of 2 proteins and acne (**Supplementary Table 9**). In addition, the results of the colocalization analysis indicate that ANXA5 exhibits strong genetic colocalization with a posterior probability of heterogeneity (PPH4) at 0.935. FSTL1 also demonstrates a trend towards colocalization (PPH4 = 0.684). These findings are detailed in **Figure 4** and **Supplementary Table 10**.

**Table 2:** Comprehensive evidence to identify drug targets in acne.

	Incident acne									
Protein	Cohort analyse		Genetic analyse			Expression	Drug Development			
	HR (95%CI)	p-value/FDR	TSMR		SMR	ScRNA-seq & serum protein	Drug Name	Outcomes	Actions	Trial Phase
			OR (95%CI)	p-value						
**ANXA5**	1.32 [1.12;1.56]	8.76e-06/0.00257	2.20 [1.32;3.65]	0.00233	**√**	**√**	/	/	/	/
**FSTL1**	1.24 [1.02;1.52]	2.93e-06/0.00171	1.53 [1.09;2.15]	0.01464	**√**	**√**	/	/	/	/

HR was adjusted by BMI (kilograms per square metre, continuous), age (sequential), sex (male or female), Townsend Deprivation Index (continuous), average annual household gross income (<£18,000, £18,000- £30,999, £31,000- £51,999, £52,000- £100,000, >£100,000, And “not known” or missing), education (CSEs or equivalent, A levels/AS levels or equivalent, College or University degree, NVQ or HND or HNC or equivalent, O levels/GCSEs or equivalent, professional qualifications, and None of the above), the season of blood collection, smoking status (current, previous or never), alcohol consumption (daily, month to week or never), physical activity (met-minutes per week, Consecutive).

HR, hard ratio; CI, confidence interval.

### Phenome-wide MR analysis

Given the pivotal role drugs play through the bloodstream, our study explored the influence of 2 blood protein expressions on various health conditions. We undertook a comprehensive MR analysis across 1,402 conditions and traits cataloged in the UK Biobank. Our results revealed no significant associations (referenced in **Supplementary Figure 2**).

### Differential expression analysis and cell-type specificity expression in the skin tissue

**Figure 5:**
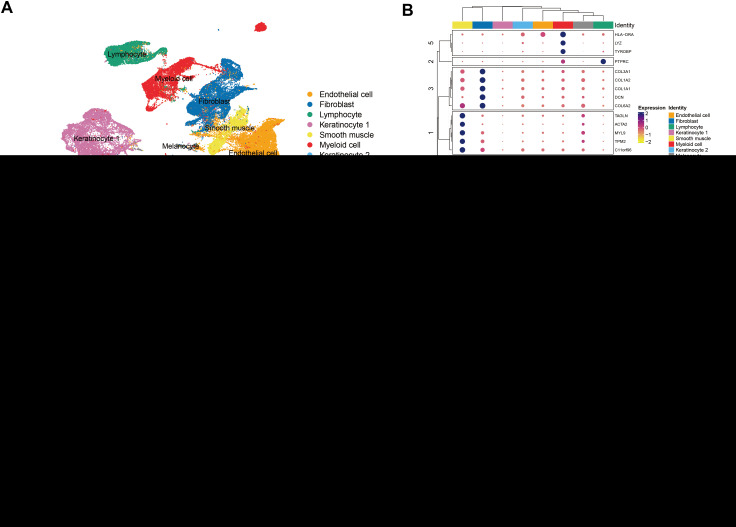
Single-Cell Transcriptomic Exploration of Acne Drug Targets. **(A)** UMAP Clustering Overview: Depicts a UMAP dimensionality reduction map of the acne single-cell transcriptome dataset. Each point signifies an individual cell, with varying colors highlighting distinct cellular clusters. **(B)** Cell Marker Clustering Heatmap: This heatmap delineates cell grouping based on marker expression. Different color modules represent various cell groups, and the circle size reflects the expression proportion of target genes within these specific groups. **(C)** Gene Feature Plot for Acne Targets: Exhibits the expression dynamics of genes linked to two primary acne targets. In this visualization, each point corresponds to a cell, and the color intensity denotes the expression level, with darker shades indicating higher expression. **(D)** Violin Plots for Gene Expression Disparities: Showcases the expression variation of genes associated with the two acne targets across skin lesions versus non-lesioned skin of the dermis and epidermis. Violin plots are used for representation, with black inverted triangles marking the average expression values. * means P value less than 0.05, **** means P value less than 0.0001.

The protein group differential analysis indicated that, For acne, the serum levels of FSTL1 and ANXA5 were significantly elevated in patients relative to the control group. (**Supplementary Figure 3**). To ascertain if FSTL1 and ANXA5 exhibited cell type-specific enrichment in dermatological tissues, we conducted a single-cell type expression analysis using scRNA-seq data from the Gene Expression Omnibus (GEO). A total of 8 cell types were identified in the acne dataset (**Figure 5A**), and markers of specific expression of each cell were presented in **Figure 5B**. ANXA5 and FSTL1 were expressed in multiple skin cells, but FSTL1 was relatively high in fibroblasts and ANXA5 was relatively high in fibroblasts and myeloid cells (**Figure 5C**). Further RNA-Seq differential analysis found that the expression of ANXA5 and FSTL1 in acne dermal lesions was significantly higher than in non-lesion sites (**Figure 5D**). However, the expression of ANXA5 and FSTL1 in acne epidermal lesions was significantly lower than in non-lesion sites. In terms of assessing druggability, no protein has been identified as a target in drug development initiatives.

## Discussion

Utilizing the UKB-PPP cohort, we integrated proteomic and genomic data to explore the longitudinal associations and causal relationships between the proteome and acne. This comprehensive analysis identified two potential therapeutic targets for acne. Notably, these proteins have not yet been targeted in drug development efforts. Further validation through cell-specific expression verification, conducted via single-cell transcriptomics, reinforced the significance of these proteins in the pathophysiology of acne. These findings illuminate their potential utility as novel drug targets for the treatment of acne.

FSTL1 (Follistatin Like 1), a secreted glycoprotein, plays critical roles in various physiological processes including angiogenesis, regulation of the immune response, and cellular proliferation and differentiation (37). ANXA5 (Annexin A5) acts as an inhibitor of phospholipase A2 and protein kinase C, with calcium channel activity, implicating it in cell signal transduction, inflammation, and the processes of growth and differentiation. To date, the relationship between FSTL1, ANXA5, and acne has not been extensively explored in the scientific literature. Our study reveals that the expression levels of FSTL1 and ANXA5 in the transcriptome at acne dermal lesions significantly exceed those in the normal control group. This trend is mirrored in the expression levels of these proteins in the blood, suggesting a systemic component to their involvement in acne pathophysiology. Furthermore, cohort study results indicate a substantial impact on acne risk, with every one SD increase in the expression of FSTL1 and ANXA5 proteins associated with a 24% and 32% increase in the incidence of acne, respectively. These findings spotlight FSTL1 and ANXA5 as significant contributors to the development of acne, marking them as potential targets for future research and therapeutic intervention. Their elevated expression in both the dermal lesions and blood of acne patients underscores their role in acne’s pathogenesis and suggests that they may serve as biomarkers for the disease or targets for novel acne treatments.

This study’s robustness lies in its integrative approach, combining the proteome with the genome to systematically explore the relationship between plasma protein biomarkers and the risk of acne through cohort analysis and MR. The design benefits from a substantial sample size, extensive proteome coverage, and the ability to address reverse causation with a minimal risk of confounding, ensuring the reliability of our findings. The consistency observed across various rigorous analyses further attests to the strength of our results. Nonetheless, this study is not without limitations that merit consideration. First, the study’s cohort predominantly consists of white participants, limiting the generalizability of the findings to other racial and ethnic groups. Second, the study does not measure the levels of these proteins directly within skin lesion tissues. To partly address this gap, we employed single-cell RNA sequencing to assess the gene expression levels of associated proteins in the lesions of the diseases in question. Finally, not every protein identified in our preliminary cohort analysis was backed by strong cis-pQTL, which limited our capacity to perform comprehensive cis-MR analyses for all proteins. Moreover, the efficacy of our genetic instruments varied, with those harboring a greater number of variants more likely to identify statistically significant associations. This variance in instrument strength could potentially lead to the underestimation of associations for proteins represented by fewer genetic variants.

## Conclusion

Leveraging a substantial cohort of roughly 53,000 individuals, our investigation conducted an extensive trio of genotypic, proteomic, and longitudinal clinical assessments to elucidate the circulating proteome associated with acne. This exploration successfully identified FSTL1 and ANXA5 as the potential protein targets for acne treatment. The novelty of these findings underscores the significant untapped potential within the circulating proteome for therapeutic intervention in acne.

## Data Availability

The datasets presented in this study can be found in online repositories. The names of the repository/repositories and accession number(s) can be found in the article/**Supplementary Material**.
