# Supplementary material for: Unveiling new protein biomarkers and therapeutic targets for acne through integrated analysis of human plasma proteomics and genomics
Source: Front Immunol. 2024 Oct 18;15:1452801. doi: 10.3389/fimmu.2024.1452801 (PMC11527721; doi:10.3389/fimmu.2024.1452801)
Supplement: Supplementary file 1 [file DataSheet1.docx]

**Supplement 2**

EMethods

eFigure 1. The Theoretical Underpinnings and Principal Assumptions of Mendelian Randomization.

eFigure 2. Phenome-Wide Association Insights via Mendelian Randomization: Implications of Two Acne-Related Drug Targets on Disease Spectrum.

eFigure 3. Comparative Serum Protein Expression Analysis of Drug Targets in Acne.

eMethods

**SMR analysis and Heterogeneity in Dependent Instruments (HEIDI) test**

In employing pQTL for research, the SMR method is utilized to derive effect estimates. This approach capitalizes on aggregated data from Genome-Wide Association Studies (GWAS) and pQTL studies to investigate the relationships between protein expression levels and specific health outcomes(1). Analysis and allele coordination were executed using the SMR software, version 1.3.1, available at [https://yanglab.westlake.edu.cn/software/smr/]. To amalgamate the effect estimates, an IVW-MR strategy was adopted. The HEIDI test, integral to the SMR framework, was employed to determine if the gene expression-outcome associations could be attributed to linkage scenarios. This is discerned when the HEIDI test produces a p-value of less than 0.01, suggesting a possible linkage origin for the association(2).

**Bayesian co-localization analysis**

To evaluate whether two traits—protein expression levels and phenoage—are influenced by identical causal genetic variants, we employed Bayesian co-localization analysis using the 'coloc' package(3), accessible at https://github.com/chr1swallace/coloc. This method calculates posterior probabilities for five distinct hypotheses regarding the potential overlap of genetic variants between the proteome and phenoage. Our analysis particularly focused on the posterior probability of hypothesis 3 (PPH3) and hypothesis 4 (PPH4). PPH3 suggests that the protein expression and phenoage are influenced by separate genetic variants within the same region, while PPH4 indicates a shared genetic basis for both traits in the region. To determine evidence of co-localization, we utilized the coloc.abf and coloc.susie algorithms. A gene was considered to show co-localization if it demonstrated a gene-based PPH4 greater than 80%, as confirmed by at least one of the algorithms.

**Phenome-Wide MR Analysis**

To investigate potential adverse reactions associated with the target druggable gene, we conducted phenome-wide MR analyses. In this process, pQTL data related to the druggable gene served as the exposure variable. This data was contrasted with comprehensive GWAS data on a variety of diseases documented within the UK Biobank cohort (n ≤ 408,961), which acted as the outcome variable. Lee et al. have performed GWAS analyses on multiple diseases within the UK Biobank, utilizing the SAIGE (V.0.29) methodology(4,5). This approach, known for its adept extension and accurate application of generalized mixed models, effectively navigates the complexities of imbalanced case-control ratios. For our phenome-wide MR explorations, we focused on 1,402 traits, each representing a unique disease with no fewer than 50 reported cases. We sourced composite statistics for disease-associated SNPs from the SAIGE GWAS database (available at: https://www.leelabsg.org/resources), with further methodological details outlined in their publication. The Wald ratio method was applied to perform MR analyses, ensuring a uniform approach to parameter application. A stringent significance threshold of P < 0.05/1,402 was set to identify causally significant associations.

**Single-cell RNA Sequencing Data Analysis**

Single-cell RNA sequencing (scRNA-seq) datasets of acne was sourced from publicly accessible databases(6). The specifics of each dataset, including their origins and characteristics, are detailed in eTable 4 of **supplement 1**. For the analysis of these datasets, we employed the Seurat software package, initiating the process with stringent quality control criteria. This involved selecting cells that exhibited a range of nFeature counts from 200 to 5000 and contained less than 25% mitochondrial RNA content, ensuring the exclusion of low-quality or dying cells. Following quality control, dataset integration was performed using the Harmony package, a method chosen for its efficacy in harmonizing data from different sources while retaining biological variability. The integrated datasets then underwent normalization to correct for technical variations, and dimensionality was reduced using Unified Manifold Approximation and Projection (UMAP). This step facilitated the visualization of complex data in a lower-dimensional space, enhancing the identification of cellular clusters. Subsequent clustering allowed for the categorization of cells into groups based on their gene expression profiles. These groups were then annotated with cell-specific markers, enabling the identification of distinct cell populations within the datasets.

References

1. Zhu Z, Zhang F, Hu H, Bakshi A, Robinson MR, Powell JE, et al. Integration of summary data from GWAS and eQTL studies predicts complex trait gene targets. Nat Genet. 2016 May;48(5):481–7.

2. Chauquet S, Zhu Z, O’Donovan MC, Walters JTR, Wray NR, Shah S. Association of Antihypertensive Drug Target Genes With Psychiatric Disorders: A Mendelian Randomization Study. JAMA Psychiatry. 2021 Jun 1;78(6):623–31.

3. Wang G, Sarkar A, Carbonetto P, Stephens M. A simple new approach to variable selection in regression, with application to genetic fine mapping. J R Stat Soc Series B Stat Methodol. 2020 Dec;82(5):1273–300.

4. Zhou W, Nielsen JB, Fritsche LG, Dey R, Gabrielsen ME, Wolford BN, et al. Efficiently controlling for case-control imbalance and sample relatedness in large-scale genetic association studies. Nat Genet. 2018 Sep;50(9):1335–41.

5. Zhou W, Bi W, Zhao Z, Dey KK, Jagadeesh KA, Karczewski KJ, et al. SAIGE-GENE+ improves the efficiency and accuracy of set-based rare variant association tests. Nat Genet. 2022 Oct;54(10):1466–9.

6. Do TH, Ma F, Andrade PR, Teles R, de Andrade Silva BJ, Hu C, et al. TREM2 macrophages induced by human lipids drive inflammation in acne lesions. Sci Immunol. 2022 Jul 22;7(73):eabo2787.


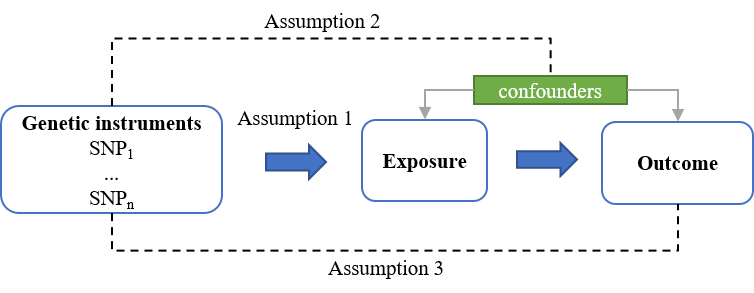


eFigure 1: The Theoretical Underpinnings and Principal Assumptions of Mendelian Randomization. This figure outlines the conceptual framework foundational to Mendelian Randomization (MR) studies, delineating its core assumptions critical for valid causal inference. Assumption 1 mandates that the genetic variant demonstrates a strong association with the exposure of interest. Assumption 2 requires that the instrumental genetic variants are not influenced by confounders that could affect the relationship between the exposure and outcome. Finally, Assumption 3 stipulates that the genetic variants impact the outcome solely through their effect on the exposure, ensuring a direct causal pathway.


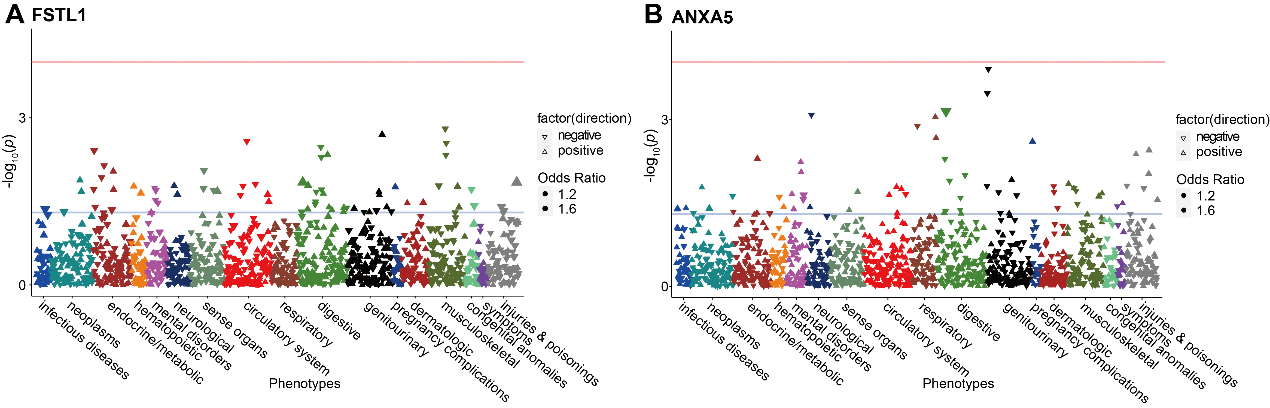


eFigure 2. Phenome-Wide Association Insights via Mendelian Randomization: The Role of Two Acne-Associated Drug Targets Across a Disease Spectrum. This Manhattan plot is derived from an extensive phenome-wide Mendelian Randomization (MR) analysis, focused on evaluating the influence of two acne-related blood expression drug targets (FSTL1 and ANXA5) over a wide range of 1,402 diseases as documented in the UK Biobank (UKB). The y-axis is a detailed record of the p-values obtained from the phenome-wide MR investigation. A gray line across the plot sets the conventional significance threshold at p < 0.05, whereas a pink line introduces an adjusted significance threshold at p < 3.56e-5, which accounts for the multiple comparisons conducted across 1,402 diseases. The orientation of each triangle marker on the plot—pointing upward for a positive effect and downward for a negative effect—graphically represents the directional causal impact that these specific blood expression drug targets have on diverse diseases.


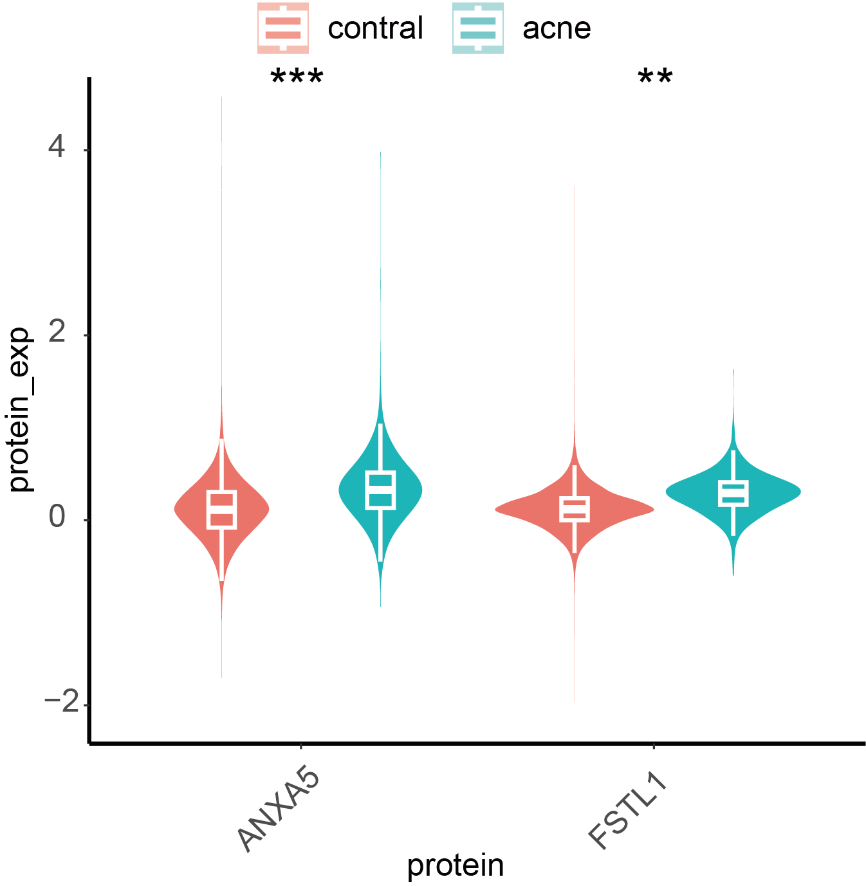


eFigure 3: Comparative Serum Protein Expression Analysis of Drug Targets In Acne. This figure utilizes violin plots to depict the differential expression levels of serum proteins associated with five drug targets in acne. The color coding distinguishes between the normal control group (red) and patients with acne. Statistical significance is annotated as follows: ns denotes no significant difference; one asterisk (*) indicates p < 0.05; two asterisks (**) denote p < 0.01; three asterisks (***) represent p < 0.001; and four asterisks (****) signal p < 0.0001.
